# Supplementary material for: Novel Therapies to Address Unmet Needs in ITP
Source: Pharmaceuticals (Basel). 2022 Jun 23;15(7):779. doi: 10.3390/ph15070779 (PMC9318546; doi:10.3390/ph15070779)
Supplement: Supplementary file 1 [file pharmaceuticals-15-00779-s001.zip › pharmaceuticals-1659111-supplementary.pdf]

SUPPLEMENTARY FIGURES AND TABLES

Supplementary Figure S1. Targets of the first and second-line treatments in ITP.
APC, antigen presenting cell; CTC, corticosteroids; ITP, primary immune thrombocytopenia; KC, Kupffer cell; RTX, rituximab; TPO-RAs, thrombopoietin receptor agonists.

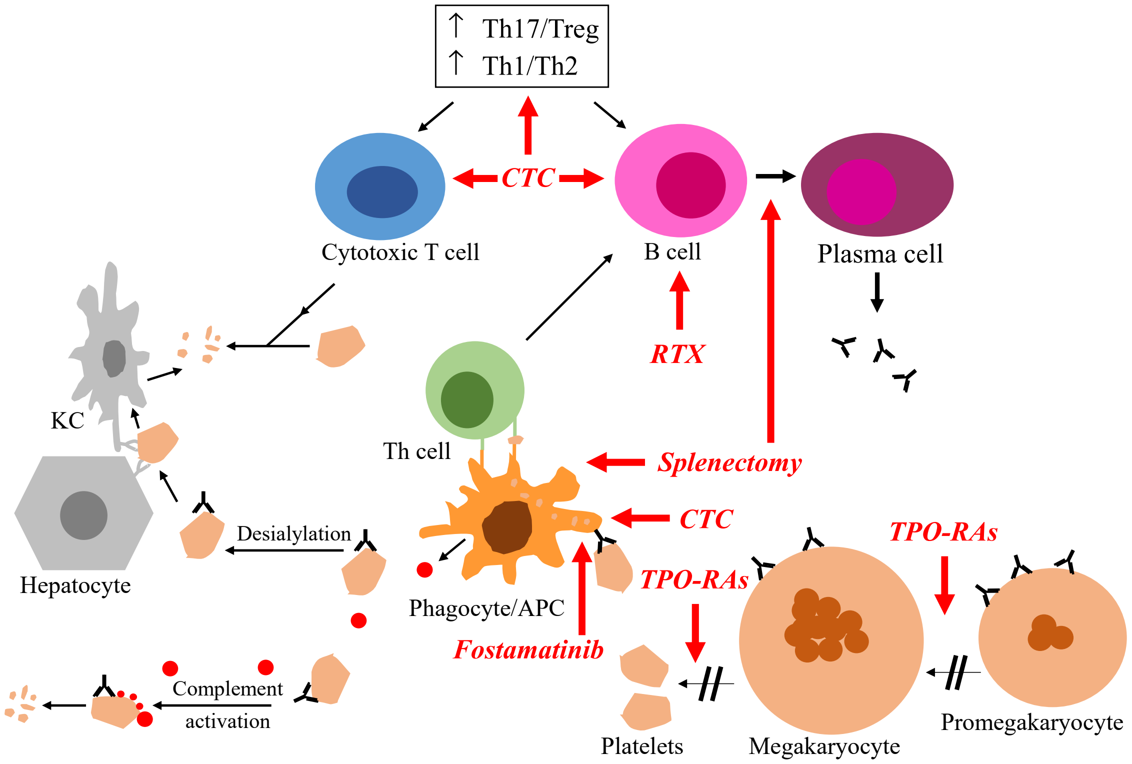

Supplementary Figure S2. Targets of the newer ITP treatments.
APC, antigen presenting cell; ITP, primary immune thrombocytopenia; KC, Kupffer cell.

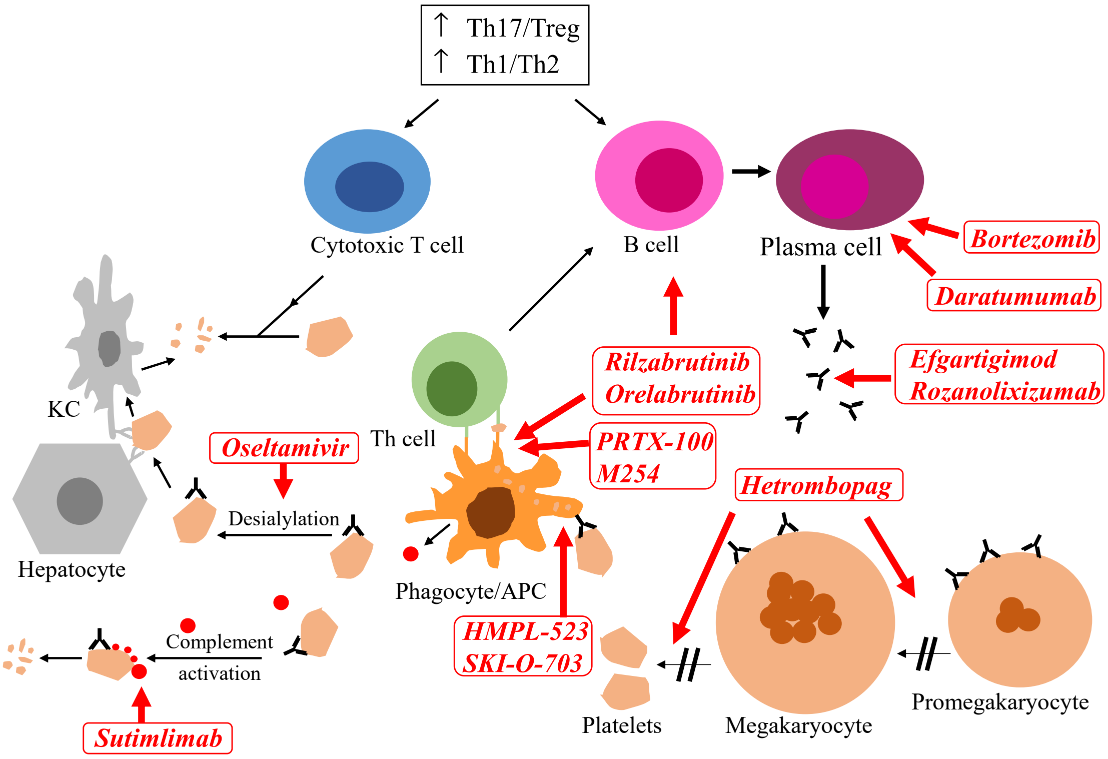

Supplementary Figure S3. Inhibition of Syk. HMPL-523 and SKI-O-703 block the kinase activity of Syk, which is engaged soon after interaction between the Fc domain of the platelet-opsonizing autoantibody and the receptor for Fc domain on the immune cell. Blocking of Syk prevents downstream signaling, thus reducing cell phagocytic capacity.
Syk, spleen tyrosine kinase.

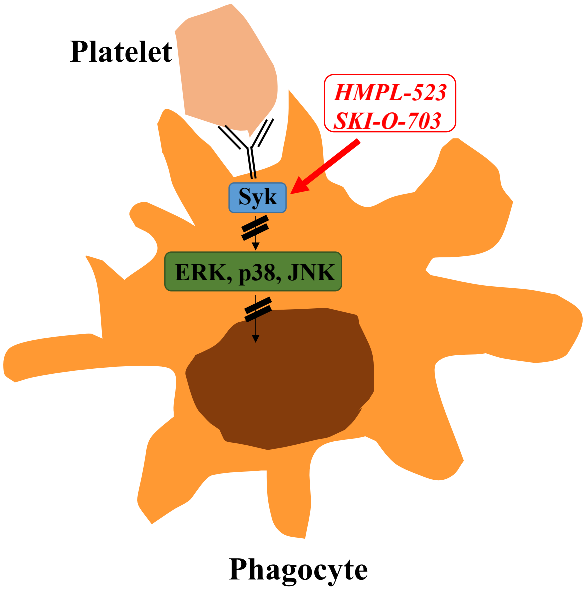

Supplementary Figure S4. Inhibition of BTK. Rilzabrutinib and orelabrutinib block the kinase activity of BTK, which plays a central role in the signaling pathways triggered upon interaction between the Fc domain of the platelet-opsonizing autoantibody and the receptor for Fc domain on the immune cell. As a consequence, the activity of various cell actors responsible for the immune disbalance in ITP is reduced. BTK, Bruton's tyrosine kinase; Syk, spleen tyrosine kinase.

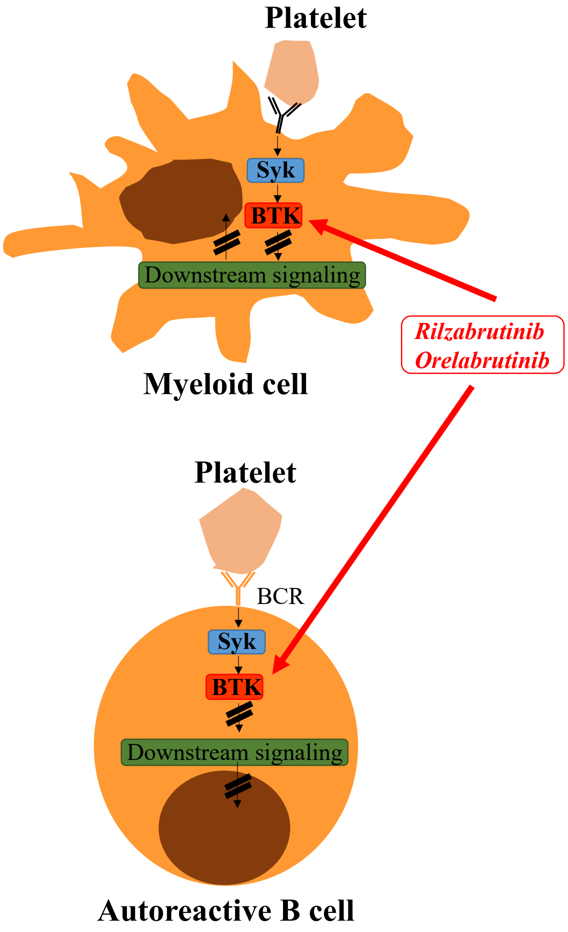

Supplementary Figure S5. Inhibition of FcRn. Circulating antibodies experience a process of cell recycling, during which they are embedded in endosomes. Binding to FcRn prevents release of antibodies into the lysosomal compartment, and its subsequent cleavage, allowing the release to the extracellular fluid. Prevention of the interaction between antibodies and FcRn (efgartigimod, rozanolixizumab) facilitates antibody destruction.
FcRn, neonatal Fc receptor.

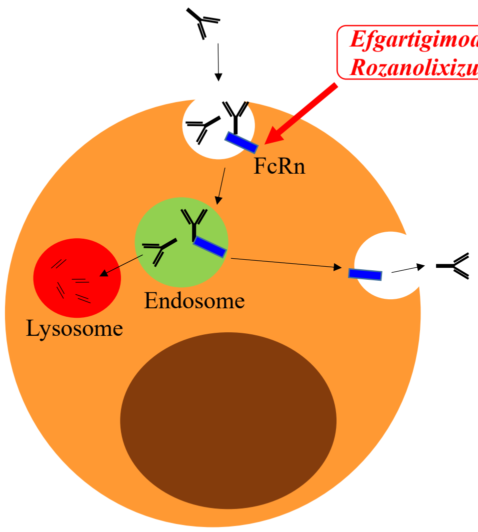

Circulating and endothelial cells

Supplementary Figure S6. Direct inhibition of FcγRIII. Opsonized platelets are fixed to the phagocyte surface by the Fc domain of the autoantibody, which interacts with membrane glycoprotein FcγRIII (CD16). Staphylococcal protein A (PRTX-100) is able to inhibit FcγRIII (CD16), thus disrupting the process leading to destruction of the opsonized platelet.

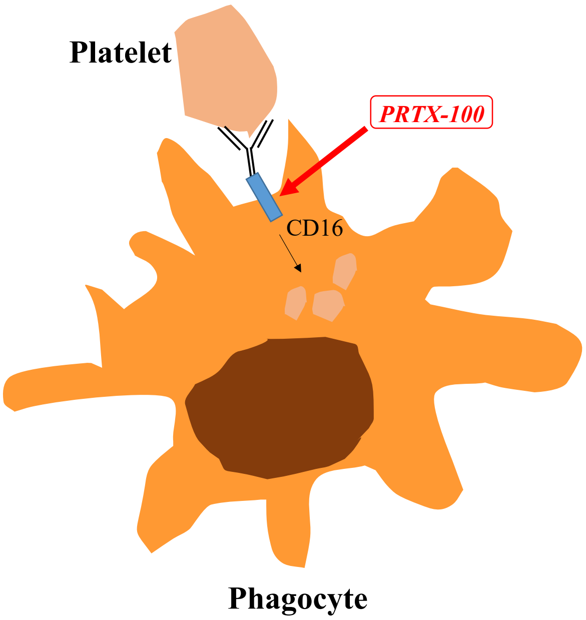

Supplementary Figure S7. Inhibition of FcγRIII by promoting FcγRIIb activity. Unlike many Fc receptors of the phagocyte, FcγRIIb exerts antiphagocytic activity by inhibiting FcγRIII. Hyper-sialylated immunoglobulins (M254) promote efficiently up-regulation of the FcγRIIb.

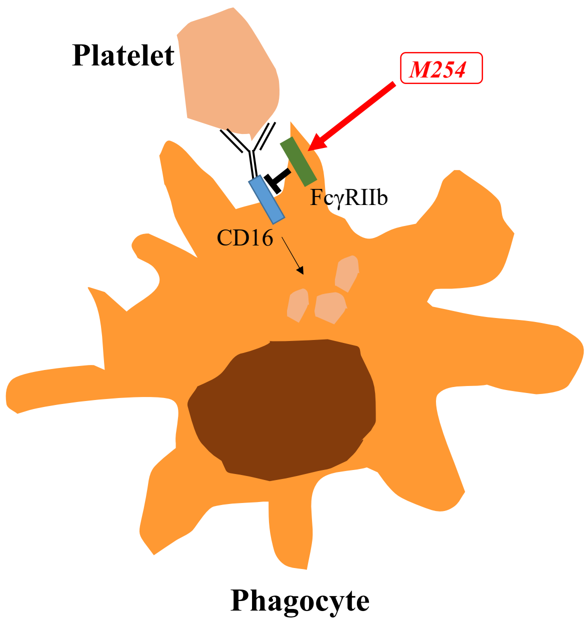

Supplementary Figure S8. Inhibition of the classical pathway of the complement system. The Fc domain of opsonizing autoantibodies triggers activation of complement by the classical pathway. By blocking the activity of the C1s component of complement system, sutimlimab induces the disruption of the cascade and the subsequent assembly of the membrane attack complex.

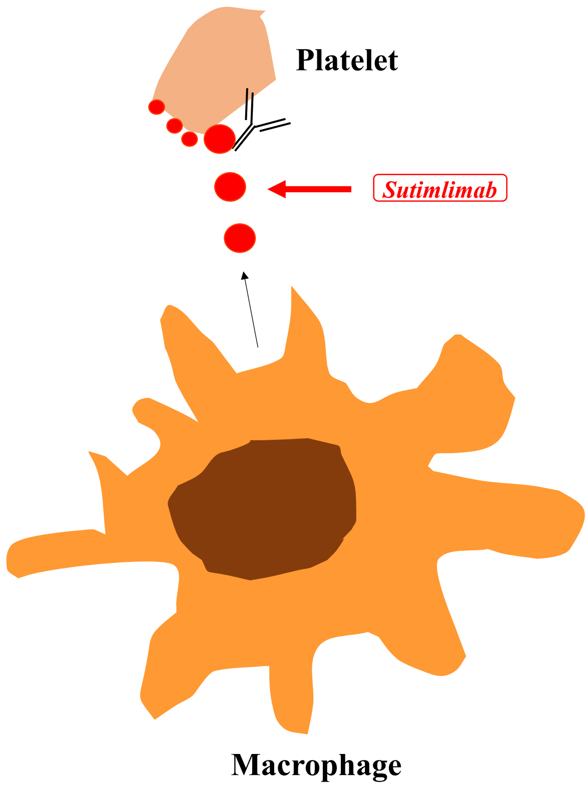

Supplementary Figure S9. Inhibition of platelet desialylation. Opsonized platelets are not protected against desialylation of O-glycans of surface glycoproteins by neuraminidases, and desialylated platelets are cleared in the liver through a coordinated action between hepatocytes, which react with desialylated platelets via Ashwell-Morell receptors, and Kupffer cells. By blocking neuraminidase activity, oseltamivir prevents platelet desialylation and the subsequent hepatic clearance.
KC, Kupffer cells.

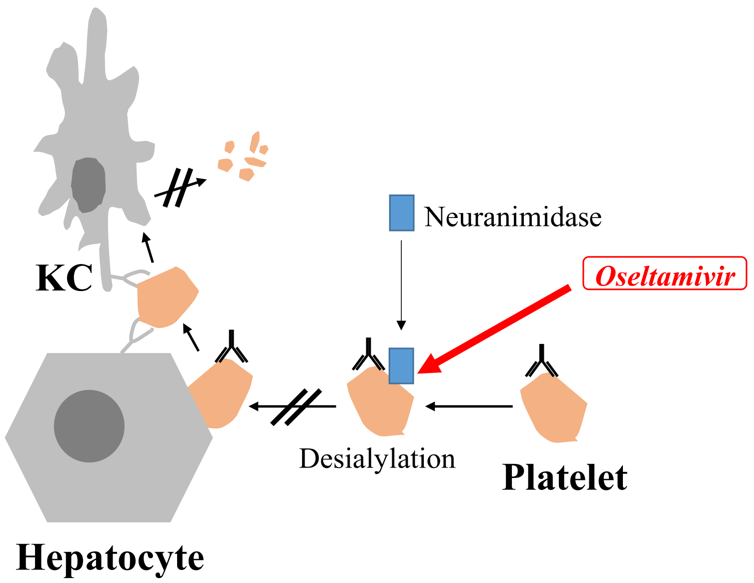

Supplementary Figure S10. Inhibition of CD38. CD38 is expressed in a variety of immune cells, and particularly on the surface of antibody-producing plasmablasts, where it exerts prosurvival and other actions. By inhibiting CD38, daratumumab reduces production of autoantibodies and cytokines by plasma cells and NK respectively, and promotes apoptosis.
NK, natural killer cells.

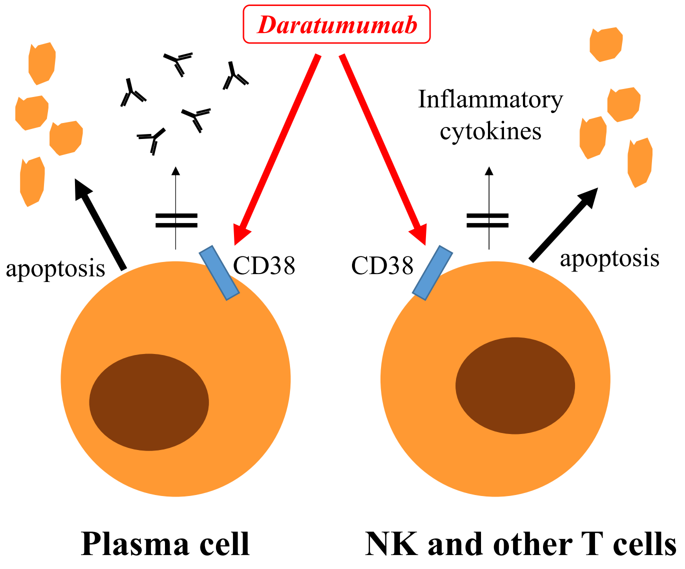

Supplementary Figure S11. Inhibition of the proteasome. The proteasome plays an important role in survival by preventing accumulation of misfolded and damaged proteins. Inhibiting proteasome activity by bortezomib can reduce the viability of plasma cells, thus avoiding the release of autoantibodies.

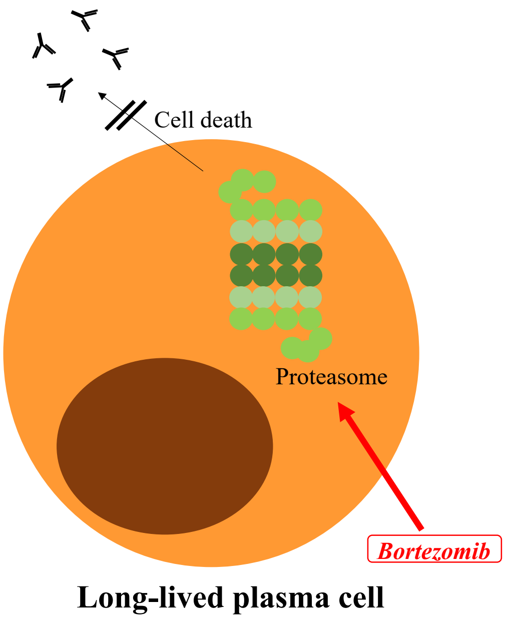

Supplementary Table S1. Features of TPO-RAs

Topic	Romiplostim	Eltrombopag	Avatrombopag
Molecular structure	Peptide	Small molecule	Small molecule
TPO-R binding	Extracellular domain	Transmembrane domain	Transmembrane domain
JAK/Stat activation	++++	+	+
Route of administration	Subcutaneous	Oral	Oral
Dosing frequency[a]	Weekly	Daily[b]	Daily
Relevant food interactions	n.a.	Yes (polyvalent cations)	No
Lower dose if liver dysfunction	No	Yes	No
Use in renal failure	Yes	Probably yes	Probably yes
Use in pregnancy	No	No	No
Current indications	Chronic ITP (adults and children)	Chronic ITP (adults and children)<br>Hepatitis C-associated thrombocytopenia<br>Severe aplastic anemia	Chronic ITP (adults)<br>Periprocedural thrombocytopenia in CLD

[a]Per drug label. [b]May sometimes be given less frequently. CLD, chronic liver disease; ITP, primary immune thrombocytopenia; n.a., not applicable; TPO-R, thrombopoietin receptor; TPO-RAs, thrombopoietin receptor agonists.

Supplementary Table S2. Characteristics of drugs used as third (or more) line options in ITP management

Drug	Dose	Onset of action/ Peak response (d)	Overall response	Side effects
**MMF**	500 mg p.o. BD for 2 wk, then 1 g p.o. BD	28-56/n.a.	30-60%	Diarrhea, nausea, headache, teratogenicity
**Cyclosporin A**	2.5-3 mg/kg/d p.o., titrating to keep level in 100-200 ng/mL	21-28/n.a.	Complete response: 40% Platelet improvement: 50-80%	Hypertension, renal dysfunction, hypertrichosis, gum hypertrophy
**Danazol**	400-800 mg/d p.o.	14-90/28-180	30-60%	Acne, hirsutism, amenorrhea, liver dysfunction
**Azathioprine**	1-2 mg/kg/d p.o. (maximum 150 mg)	3-90/30-180	30-60%	Fatigue, neutropenia, hepatotoxicity, increased risk of malignancy
**Dapsone**	75-100 mg/d p.o.	21-28/n.a.	30-60%	Nausea, dyspepsia, methemoglobline mia, skin rash, hemolysis if G6PD deficiency

BD, twice a day; d, days; G6PD, glucose-6-phosphate dehydrogenase; MMF, mycophenolate mofetil; n.a., not available; p.o., oral; wk, weeks.
